# Mineral Particles in Foliar Fertilizer Formulations Can Improve the Rate of Foliar Uptake

**DOI:** 10.3390/plants13010071

**Published:** 2023-12-25

**Authors:** Carlos Pimentel, Carlos M. Pina, Nora Müller, Luis Adrián Lara, Gabriela Melo Rodriguez, Fabrizio Orlando, Joachim Schoelkopf, Victoria Fernández

**Affiliations:** 1 Université Grenoble Alpes, Université Savoie Mont Blanc, CNRS, IRD, Université Gustave Eiffel, ISTerre, 38000 Grenoble, France; 2 Departamento de Mineralogía y Petrología, Facultad de Ciencias Geológicas, Universidad Complutense de Madrid, 28040 Madrid, Spain; cmpina@geo.ucm.es; 3 Instituto de Geociencias (UCM-CSIC), 28040 Madrid, Spain; 4 New Applications Research Group, Research and Development Services, Omya International AG, 4622 Egerkingen, Switzerland; nora.mueller@omya.com (N.M.); gabriela.melorodriguez@omya.com (G.M.R.); fabrizio.orlando@omya.com (F.O.); joachim.schoelkopf@omya.com (J.S.); 5 Systems and Natural Resources Department, School of Forest Engineering, Polytechnic University of Madrid, 28040 Madrid, Spain; ladrianlaro@gmail.com; 6 Centro para la Conservación de la Biodiversidad y el Desarrollo Sostenible, School of Forest Engineering, Polytechnic University of Madrid, 28040 Madrid, Spain

**Keywords:** adjuvants, carbonate minerals, foliar uptake, foliar fertilizers, geochemical modelling

## Abstract

The application of foliar sprays of suspensions of relatively insoluble essential element salts is gradually becoming common, chiefly with the introduction of nano-technology approaches in agriculture. However, there is controversy about the effectiveness of such sparingly soluble nutrient sources as foliar fertilizers. In this work, we focussed on analysing the effect of adding Ca-carbonate (calcite, CaCO_3_) micro- and nano-particles as model sparingly soluble mineral compounds to foliar fertilizer formulations in terms of increasing the rate of foliar absorption. For these purposes, we carried out short-term foliar application experiments by treating leaves of species with variable surface features and wettability rates. The leaf absorption efficacy of foliar formulations containing a surfactant and model soluble nutrient sources, namely Ca-chloride (CaCl_2_), magnesium sulphate (MgSO_4_), potassium nitrate (KNO_3_), or zinc sulphate (ZnSO_4_), was evaluated alone or after addition of calcite particles. In general, the combination of the Ca-carbonate particles with an essential element salt had a synergistic effect and improved the absorption of Ca and the nutrient element provided. In light of the positive effects of using calcite particles as foliar formulation adjuvants, dolomite nano- and micro-particles were also tested as foliar formulation additives, and the results were also positive in terms of increasing foliar uptake. The observed nutrient element foliar absorption efficacy can be partially explained by geochemical modelling, which enabled us to predict how these formulations will perform at least in chemical terms. Our results show the major potential of adding mineral particles as foliar formulation additives, but the associated mechanisms of action and possible additional benefits to plants should be characterised in future investigations.

## 1. Introduction

Foliar fertilizers are commonly used in commercial agriculture to complement root fertilization, or as an alternative means to supply nutrients to plants during the growing season, such as when plants’ nutrient demand exceeds the root absorbing capacity [1]. Many plant-related, environmental, and physio-chemical factors affect the foliar absorption process [2]. Furthermore, the leaf absorption pathways involved may vary among plant species, organs’ state of development, or environmental conditions [3]. The complexity of the scenario associated with the uptake of foliar sprays still hinders the optimisation of foliar fertilizer treatments [2].

The physio-chemical characteristics of spray formulations have been recognised as key factors affecting foliar absorption rates [4,5,6,7]. The properties of both mineral elemental compounds and formulation adjuvants (e.g., surfactants or humectants), such as point of deliquescence (or deliquescence relative humidity) [8,9], point of efflorescence (or efflorescence relative humidity) [10,11,12], solubility [7], or physiological effects [13] should be considered when designing a foliar spray formulation [2].

Over the last decade, nanotechnological approaches have been introduced for the development of fertilizers [14,15,16]. Several studies have examined the potential absorption of nano- and micro-particles of sparingly soluble mineral compounds after foliar application, e.g., [17,18,19,20,21,22,23,24].

Despite the traditional reluctance to supply sparingly soluble nutrient forms such as carbonates or oxides [7], recent studies have shown that they can be absorbed via the foliage and have great potential as slow-release fertilizers, e.g., [18,25,26,27]. For example, in a study developed with *Avena sativa* plants, Kaiser [17] reported the presence of particles in stomatal cavities after spraying the plants with a suspension of 10 g L^−1^ calcium carbonate (CaCO_3_) particles (90% (*w*/*w*) particles smaller than 2 µm and 60% < 1 µm) in addition to 0.1% Silwet Gold organosilicon surfactant. Thus, growing evidence demonstrates that foliar-applied nutrient suspensions of sparingly soluble chemical forms can be absorbed by the foliage at lower, but still significant rates compared to soluble chemical forms.

Calcium is an essential nutrient for plants, with major signalling and structural roles [28,29,30]. This element is taken up from the soil solution by the roots and is translocated via the xylem following the transpiration stream [31]. It has low phloem mobility and is accumulated in mature organs, while it is chiefly required in developing tissues [30]. Calcium is transported in the xylem from roots to shoots, and its distribution is related to plant water fluxes, so that highly transpiring organs such as leaves receive more Ca than low transpiring plant parts like fruits [32].

Given the limited translocation of Ca from leaves to fruits and within the plant during fruit growth [33,34], the application of Ca sprays to developing fruits is often recommended [35,36,37]. The absorption of foliar-applied Ca-carbonate particles may be related to the stomatal pathway when the pores are open [38], especially if surfactants are added to the foliar treatments [17]. Calcium supplied as carbonate may also penetrate through the cuticle as Ca^2+^ ions. Among other factors, the solubility of Ca-carbonate is increased under acid pH conditions [39]. Hence, once Ca-carbonate has crossed the leaf surface, it may be more easily dissolved in the apoplast, whose pH ranges between 5 and 6 [1]. The penetration of zinc (Zn), supplied as Zn-oxide, through the leaf cuticle and also through the trichomes of several plant species has been shown by Li et al. [18,25].

Many studies carried out over the past 70 years have evaluated the rate of absorption of foliar-applied nutrients, using intact leaves or isolated automatous leaf cuticles, also supplying radiolabelled compounds [2]. The highest rate of foliar absorption takes place during the drop-drying process (i.e., shortly after foliar fertilizer application) [2], but many factors may influence foliar uptake efficiency as described above. Hence, for evaluating the performance of foliar formulations, short-term leaf uptake trials are often performed [7,9,11,12]. The methodology employed in such foliar penetration investigations is directed towards estimating the amount of nutrients that are transported across the cuticle or leaf surfaces [2,3], and not towards evaluating mineral element cell uptake in plant organs, which is affected by additional factors including, for instance, long-distance transport and membrane cell transporters [1].

Currently, there is a global trend towards a transition to a circular economy that will reduce pressure on natural resources, which is also a major challenge for agriculture [39]. Nowadays, there is a need to introduce more sustainable and environmentally friendly plant production approaches that not only contribute to a circular economy, but also preserve crop yield and quality [40]. In this regard, the use of waste materials [41] and natural products that may improve the efficacy of agrochemical and fertilizer treatments, e.g., [41,42,43], may substantially contribute to a more efficient agricultural production under the frame of a circular economy [44].

Aware of the major foliar absorption enhancement that the addition of adjuvants to foliar spray formulations can induce [2], this study aimed to evaluate the potential of carbonate mineral micro- and nano-particles as foliar fertilizer additives by treating different crop plants. As a preliminary step, the geochemical characteristics of the carbonate minerals employed as adjuvants were modelled as the rate of uptake of soluble mineral element compounds (i.e., in the form of ions when they are solubilized) has been shown to be faster and more effective, e.g., in [7,18]. The following hypotheses were tested: (i) due to their limited solubility, calcite and dolomite micro- and nano-particle sprays do not significantly increase plant tissue Ca and magnesium (Mg) concentrations after foliar application, and (ii) the addition of calcite and dolomite particles combined with soluble nutrient sources can increase the leaf absorption efficacy of foliar fertilizers.

## 2. Results

### 2.1. Geochemical Modelling for Assessing the Solubility of Mineral Particles

Aqueous geochemical modelling was carried out to evaluate the potential of calcite (CaCO_3_) and dolomite (CaMg(CO_3_)_2_) nano- and micro-particles to be used as foliar fertilizer additives.

At 25 °C, the Ca concentrations in pure water in equilibrium with CO_2_ and in equilibrium with calcite and dolomite were quite low and corresponded to 0.49 mM and 0.30 mM, respectively (Table 1). When a CaCl_2_ solution was used as a solvent, the Ca concentration in the initial CaCl_2_ solution and the CaCl_2_ solution in equilibrium with calcite and CO_2_ corresponds to 151.72 mM Ca (Table 1); i.e., the dissolution of calcite in the CaCl_2_ solution only led to an increase of ~2 mM of Ca. Similarly, dolomite particles in equilibrium with a CaCl_2_ solution only released ~2 mM of Ca (from the initial 150.00 mM CaCl_2_ to the expected 151.89 mM total Ca concentration; see Table 1). However, this increase in Ca solution concentration could have led to the precipitation of small amounts of calcite, which could have reduced the Ca concentration in the solution. This implies that the Ca concentration of the dolomite and calcite water suspension was quite low and that their combination with a soluble Ca salt such as CaCl_2_ led to the corresponding low Ca increase, as demonstrated here with Ca solubility modelling (Table 1).

When the solubility of essential element compounds (i.e., KNO_3_, MgSO_4_, ZnSO_4_) was modelled in formulations containing calcite and dolomite (Table 1), it was observed that they enhanced the dissolution of such carbonate mineral particles, hence increasing the amount of total Ca in the solution. The total Ca concentration in the K-containing formulation was slightly higher than that of calcite alone in pure water (i.e., 0.76 mM Ca_Tot_), while the Ca concentration in the Mg-containing and Zn-containing formulations was two to three times higher (i.e., 1.42 mM and 1.28 mM Ca_Tot_, respectively; Table 1). In the Mg-containing formulation, the total Ca concentration was composed of the two main aqueous species Ca^2+^ (0.76 mM) and CaSO_4(aq)_ (0.66 mM). For the rest of the formulations, Ca_Tot_ can be considered equal to Ca^2+^. On the other hand, for the formulation containing calcite together with Zn, it was supersaturated with respect to smithsonite (ZnCO_3_), which may have also precipitated in the solution. In the case of the Mg-bearing formulation, hydromagnesite (Mg_5_(CO_3_)_4_(OH)_2_·4H_2_O) was supersaturated, and may have also precipitated, in those solutions that were not able to equilibrate with atmospheric CO_2_. In all other cases, the only difference between solutions in equilibrium with atmospheric CO_2_ and solutions that were not equilibrated with atmospheric CO_2_ is that the latter had a lower concentration of calcium in solution and a higher pH (Table 1). Hence, the results show that carbonate minerals are sparingly soluble, but may increase their Ca solubility from their initial ca. 0.1 mM up to 0.2–0.9 mM Ca when combined with nutrient salts. This means that the contribution of these minerals to the existing Ca^+2^ concentration of the foliar spray formulations analysed in this study was always low.

### 2.2. Foliar Uptake Efficiency of Spray Formulations Containing Carbonate Mineral Particles

Aware of their low solubility and therefore limited potential to supply soluble Ca after foliar application, we carried out three consecutive experiments to evaluate the effect of adding nano- and micro-scale carbonate mineral particles in foliar fertilizer formulations using plants with variable plant surface features as described in the Materials and Methods section. We focussed mainly on applying calcite particles, but in light of the positive effects obtained after using calcite as an adjuvant, we tested whether similar results could be obtained with dolomite mineral particles as described below.

#### Foliar Ca Absorption of Calcite Particles plus Model Essential Element Salts

A foliar application trial with two plant species with wettable leaves having trichomes (see Section 5), namely, sunflower and basil, was carried out by supplying sprays containing MicroCal or NanoCal together with key plant nutrient solutions (i.e., MgSO_4_, KNO_3_ and ZnSO_4_) (Table 2).

**Figure 1:**
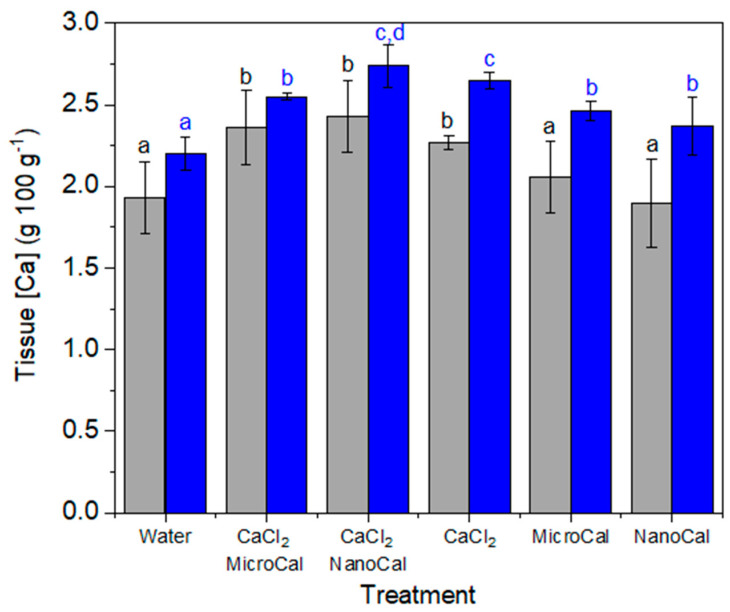
Calcium concentrations of sunflower leaves (grey bars) and basil (blue bars) aerial plant parts 1 day after foliar spraying of CaCl_2_ alone or in combination with MicroCal or NanoCal. Data are means ± SD (N = 3). Different letters indicate homogenous groups according to Tukey’s HSD test (*p* < 0.05).

**Figure 2:**
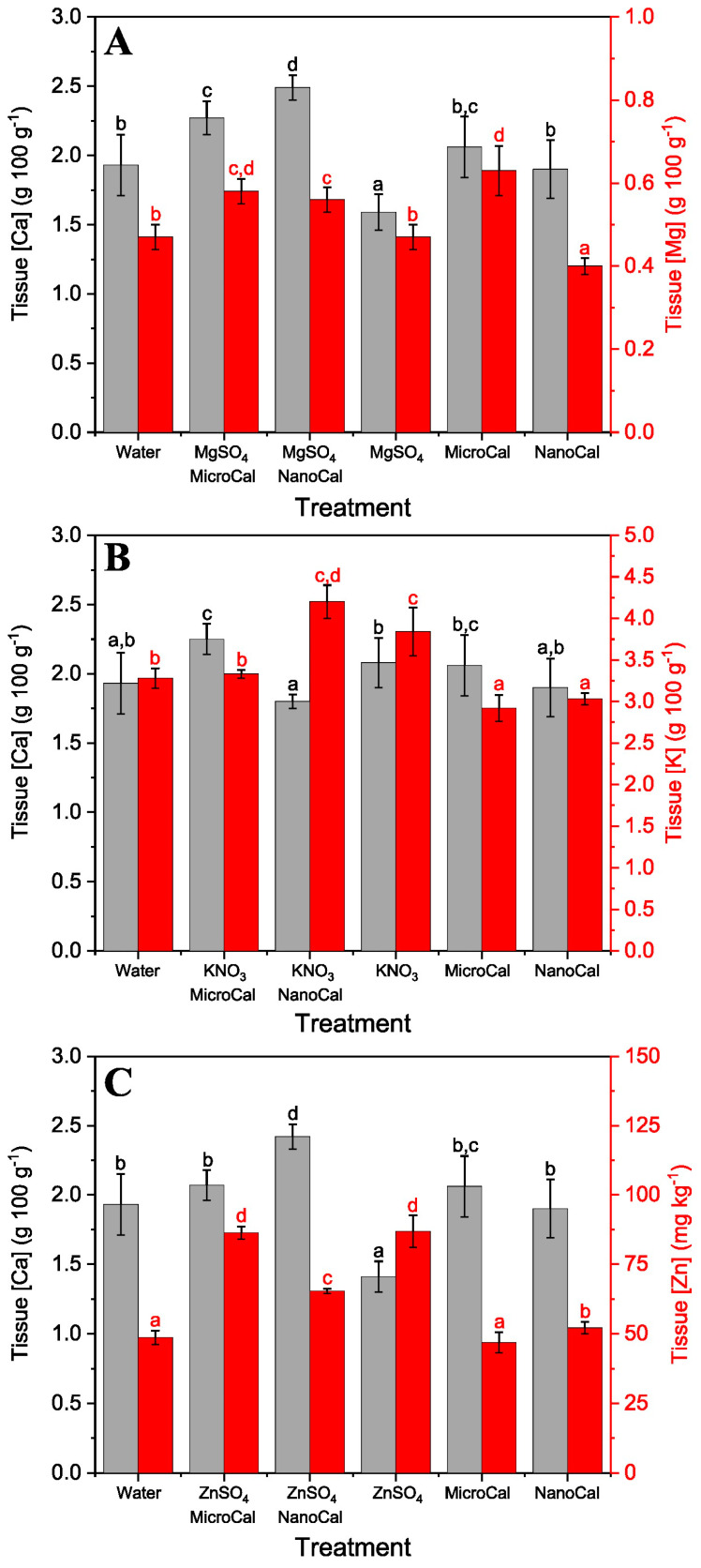
Calcium, Mg, K, and Zn concentrations of sunflower leaves 1 day after foliar spraying MgSO_4_, KNO_3,_ and ZnSO_4_ alone or in combination with MicroCal or NanoCal. Grey bars correspond to tissue Ca concentration, and red ones relate to either Mg (**A**), K (**B**), or Zn (**C**) concentrations. Data are means ± SD (N = 3). Different letters indicate homogenous groups according to Tukey’s HSD test (*p* < 0.05).

**Figure 3:**
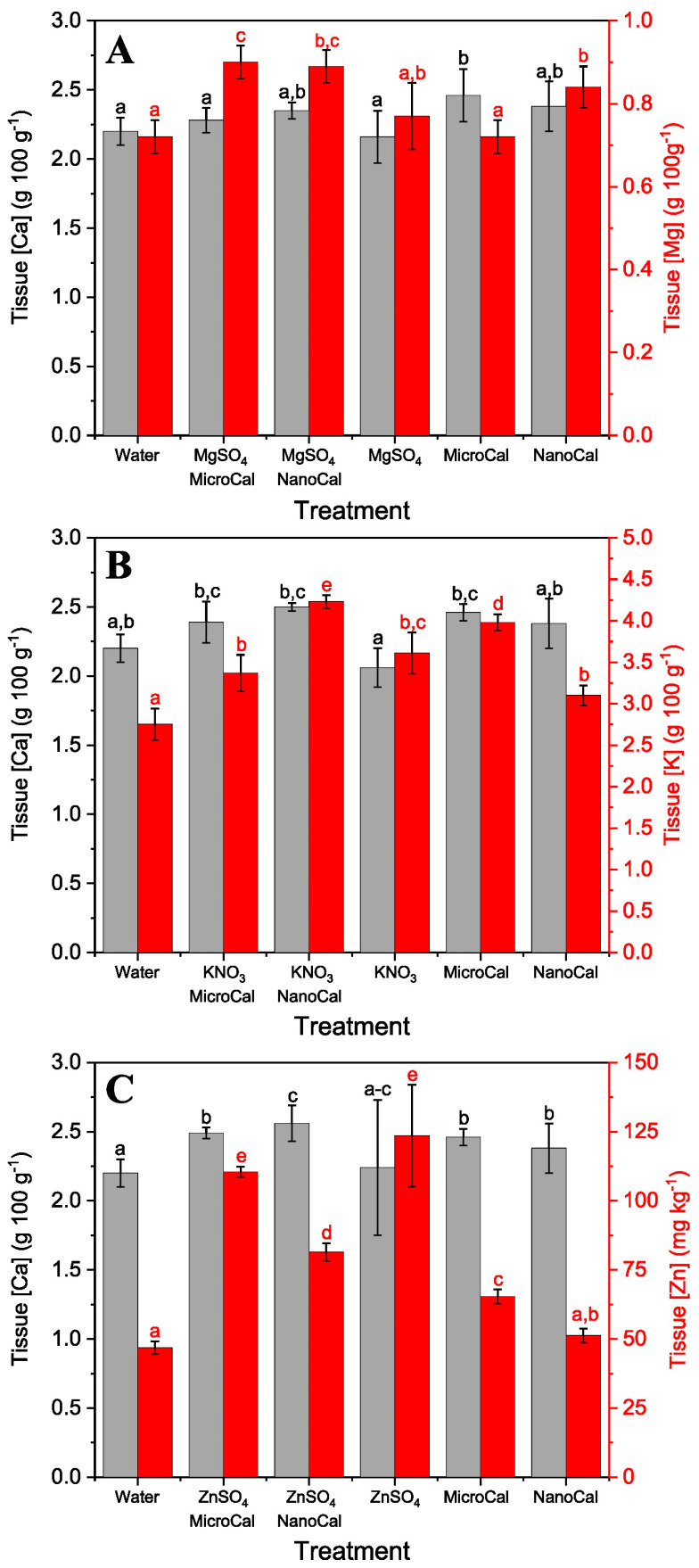
Calcium, Mg, K, and Zn concentrations of basil aerial plant parts 1 day after foliar spraying MgSO_4_, KNO_3_, and ZnSO_4_ alone or in combination or in combination with MicroCal or NanoCal. Grey bars correspond to tissue Ca concentrations, and red ones relate to either Mg (**A**), K (**B**), or Zn (**C**) tissue concentrations. Data are means ± SD (N = 3). Different letters indicate homogenous groups according to Tukey’s HSD test (*p* < 0.05).

We observed that mixing CaCl_2_ with calcite particles (MicroCal, but chiefly NanoCal; Figure 1) led to the highest tissue Ca increases in sunflower (Figure 2) and basil (Figure 3) plants. This suggests a synergistic effect of Ca-carbonate particles when supplied together with this Ca salt. In the case of these two species with wettable leaf surfaces, the application of foliar MicroCal and NanoCal alone (with a surfactant) also raised tissue Ca concentrations, but to a lesser extent compared to treatments with CaCl_2_ + calcite particles, as expected from their low solubility (Table 1).

When MgSO_4_ was mixed with calcite micro- and nano-particles, the highest Mg uptake rates were recorded in combination with both MicroCal and NanoCal (Figure 2A). Interestingly, in the case of sunflower plants, the two formulations containing MgSO_4_ + particles, especially NanoCal + MgSO_4_, also showed the highest tissue Ca concentrations. For basil leaves, the supply of MicroCal and NanoCal, mainly alone but also in combination with MgSO_4_, led to the highest tissue Ca concentrations (Figure 3A).

Formulations containing calcite particles and KNO_3_ were also sprayed onto sunflower and basil plants. For both species, the highest K uptake rate was found with the NanoCal + KNO_3_ treatment (Figure 2B). In basil, all foliar Ca and K sprays, including Ca-carbonate particles alone, increased tissue K concentrations compared to the untreated control plants (Figure 3B).

Concerning the supply of ZnSO_4_ via foliar sprays to sunflower and basil plants, the greatest increases in tissue Zn concentrations were recorded when this compound was supplied alone or in combination with calcite micro- and nano-particles (Figure 2C and Figure 3C). The rate of foliar Zn absorption was higher in basil than in sunflower plants. In general, the application of calcite particles and ZnSO_4_ increased tissue Zn and Ca concentrations (Figure 2C and Figure 3C).

### 2.3. Effect of Dolomite and Calcite Particle Foliar Application in Combination with CaCl_2_

**Figure 4:**
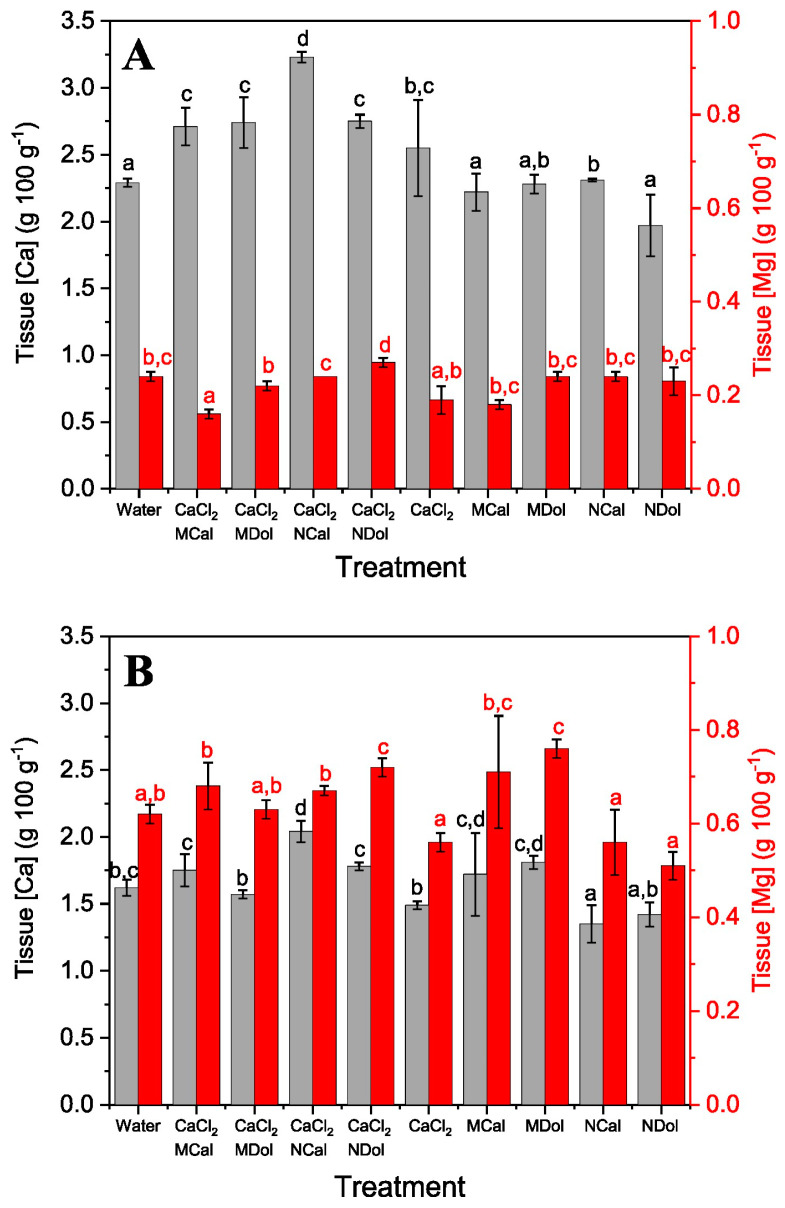
Calcium (grey bars) and Mg (red bars) concentrations of kale (**A**) and Swiss chard (**B**) aerial plant parts, 1 day after foliar spraying different treatments containing calcite and dolomite micro-and nano-particles and CaCl_2_. Data are means ± SD (N = 3). Different letters indicate homogenous groups according to Tukey’s HSD test (*p* < 0.05).

After gaining evidence for the beneficial effect of using calcite particles as foliar formulation adjuvants, we evaluated whether dolomite (CaMg(CO_3_)_2_) might also be used together with a soluble Ca fertilizer. For this purpose, the leaves of unwettable kale and wettable Swiss chard plants (see Section 5) were sprayed, and tissue Ca and Mg increases were measured as shown in Figure 4. Spray formulations containing calcite and dolomite particles plus CaCl_2_ led to significant tissue Ca increases in kale plants (Figure 4). For Swiss chard, significant tissue Mg increases were also recorded in association with dolomite particle supply (Figure 4).

Hence, both carbonate mineral particles increased the foliar uptake efficiency of CaCl_2_, especially when supplied as nanoparticles, particularly in the case of dolomite, but higher tissue Ca and Mg concentrations were also recorded after spraying Ca fertilizer formulations containing dolomite and calcite micro-particles. Once again, the application of the sparingly soluble carbonate mineral particles together with a surfactant resulted in limited to no significant increases in tissue Ca and Mg after foliar application both in Swiss chard and kale plants.

### 2.4. Foliar Ca Absorption of CaCl_2_ plus Calcite Particles: Effect of Leaf Re-Wetting

Calcite micro- and nano-particle formulations, with and without surfactant, were sprayed under controlled conditions onto the extremely unwettable cauliflower leaf surfaces (see Section 5) to assess whether it might induce a positive effect regarding potential drying–re-wetting cycles as often occur under field conditions, due to, e.g., nighttime water condensation and dew formation [11].

The highest Ca concentrations were found in leaves treated with NanoCal + CaCl_2_, followed by MicroCal + CaCl_2_ (both including surfactant; Table 2). The rate of foliar Ca absorption when CaCl_2_ alone (plus a surfactant) was applied was lower than when adding Ca-carbonate particles, again suggesting a synergistic foliar uptake effect as described above for other crop plants (Figure 1, Figure 2, Figure 3 and Figure 4).

In terms of foliar Ca absorption, the effect of re-wetting foliar-sprayed cauliflower plants via sprinkler irrigation 1 day after treatment is shown in Table 2. In general, tissue Ca concentrations were similar in foliar Ca-sprayed plants regardless of the re-wetting treatment (i.e., no re-wetting or 1 re-wetting cycle). Evidence for a positive effect of spray drop-deposit re-wetting was only gained after pure CaCl_2_ supply (i.e., including a surfactant and without calcite particles), leaf tissue Ca amounts being still below the value recorded after spraying this Ca salt plus Ca-carbonate nanoparticles (always including a surfactant). Re-wetted cauliflower plants had tissue Ca concentration values similar to those found in non-re-wetted plants (Table 2). This suggests that the Ca provided as foliar sprays was not easily washed away from the foliage by the sprinkler irrigation treatment, indicating the rain-fastness of the supplied foliar Ca formulations. It is also remarkable that the highest rates of Ca absorption were associated with nano-particle plus CaCl_2_ supply one day after treatment, indicating the suitability of performing short-term foliar absorption trials to compare the efficacy of different foliar spray formulations.

## 3. Discussion

This study focussed on assessing the potential of calcite and dolomite particles to be used as foliar formulation additives that may increase the foliar uptake rate of Ca and other essential nutrients. The two hypotheses tested proved valid, since carbonate particles supplied alone as foliar sprays led to limited Ca and Mg increases, but significantly increased the absorption of soluble nutrient salts.

Evidence for a synergistic effect when combining Ca-carbonate particles with other essential elements was gained after foliar treatment of different plant species under variable environmental conditions. Using CaCl_2_ as model nutrient element compound, we observed that such synergy also occurred after spraying fertilizer formulations containing Mg and Ca-carbonate particles (i.e., dolomite). Foliar uptake rates varied among the different plant species analysed, but an increase in the rate of foliar absorption of formulations containing calcite particles of different size was generally recorded. Plant surface composition and structure can substantially vary among plant species [3], and this may affect the rate of foliar permeability of agrochemicals [2]. The supply of foliar Ca sprays without surfactant (approx. 72 mN m^−1^ surface tension) to the extremely unwettable cauliflower plants failed to increase tissue Ca concentrations. Indeed, only few fertilizer spray drops could be observed on the surfaces which had drop repellence. Only the Ca formulations containing a surfactant led to the formation of a liquid film on the leaves, which favoured the absorption of Ca and other elements by the foliage. The surface properties (structure and composition) of sunflower, basil, Swiss chard, and kale plants will influence the foliar uptake, and future studies should try to elucidate such relationships, as described by Barlas et al. [12]. The mechanisms involved are complex and are, for example, influenced by the physicochemical properties of minerals, foliar permeability, and, ultimately, by nutrient symplastic transport [1,2].

In the following paragraphs, the significance of the results obtained is discussed with the awareness that they cannot be explained solely on the basis of the chemical composition of the foliar formulations supplied to the plants as foliar sprays. As previously mentioned, the low solubility of calcite leads to a limited release of Ca ions into the solution (only 0.49 mM in pure water at equilibrium with atmospheric CO_2_, Table 1). However, calcite dissolution can be enhanced by the addition of other chemical compounds in the formulation (e.g., KNO_3_, MgSO_4,_ and ZnSO_4_), which reduce the pH of the solutions and allow for the formation of chemical species that modify the effective solubility of calcite and dolomite.

The experiments carried out with different plant species clearly show that the supply of calcite micro- and nano-particles, together with CaCl_2_, led to the highest increases in tissue Ca. However, when these formulations were sprayed onto the foliage and plants were subsequently subjected to a drying–wetting cycle via sprinkle irrigation, an additional increase in Ca absorption due to re-wetting was only observed when CaCl_2_ was supplied alone, i.e., without Ca-carbonate particles. This performance may be associated with the higher solubility and hygroscopic properties of this Ca salt [10]. Upon re-wetting cycles, CaCl_2_ dissolves rapidly (or deliquesces due to the high humidity), releasing Ca ions into the solution that can be again taken up by the foliage [11,12]. By contrast, calcite has a low solubility, which implies that the dissolution of micro- and nano-particles is hindered, hence limiting the number of Ca ions in the solution and the potential for Ca^+2^ to be absorbed by the foliage.

The foliar penetration synergy between calcite particles and CaCl_2_ observed after foliar treatment of different species was also observed in combination with MgSO_4_ and KNO_3_. An effect of calcite particle size (i.e., nano- versus micro-particles) in terms of increased absorption of the active ingredient (i.e., Ca, Mg or K) was often measured after foliar spraying. This may be related to the higher surface area of the nano-particles, which enhances their dissolution and the release of Ca. However, many formulation aspects can influence the physio-chemical performance of Ca nano-particles, such as the interactions with other foliar spray additives. Specific trials should be carried out to elucidate this phenomenon in future investigations.

However, as shown here for ZnSO_4_, not all essential nutrient compounds may experience a synergistic foliar uptake effect when combined with Ca-carbonate particles. In our case, the addition of calcite particles reduced the amount of Zn absorbed by the foliage compared to the supply of this Zn salt alone. This reduction in foliar Zn uptake can be explained by implementing a geochemical approach as follows. Our calculations indicate that the combination of calcite particles with ZnSO_4_ in an aqueous medium can lead to the precipitation of ZnCO_3_, thus reducing the amount of Zn available in solution. This phenomenon seems to be related to particle size, as suggested by the lower Zn foliar absorption rates occurring when Ca-carbonate nano-particles were included in the foliar Zn formulations. Such foliar Zn absorption reduction can be associated with the larger surface area of Ca-carbonate nano-particles compared with that of micro-particles. In fact, the higher surface area of calcite nano-particles allows for a higher reactivity of the supplied mineral, which can result in a higher amount of precipitated ZnCO_3_.

After the development of these foliar application experiments with Ca-carbonate particles, we wondered if such foliar absorption synergy might occur after supplying carbonate mineral particles of different chemical composition. To answer this question, dolomite (CaMg(CO_3_)_2_) micro- and nano-particles were combined with CaCl_2_ and were supplied to model plant species as foliar sprays. A synergistic effect on foliar Ca absorption was also observed when employing dolomite particles as foliar formulation adjuvants (Figure 4). However, it must be noted that despite dolomite being a Ca- and Mg-containing mineral, no significant tissue Mg increases were detected in foliar-sprayed plant tissues which may be due to the particle concentration range used and other factors, such as the formation of Mg complexes that could prevent the absorption of this element by the foliage.

The synergistic effect of calcite (nano- and micro-) particles with CaCl_2_, MgSO_4_, and KNO_3_ may be partially associated with humectancy and the retention of water around the particles for a longer time compared to the supply of pure salt solutions. However, as derived from the results obtained after re-wetting of cauliflower plants previously sprayed with CaCl_2_-containing formulations, the point of efflorescence and deliquescence of active ingredients (in this case, the essential element sources supplied as foliar sprays) may also play a role [10,11].

Based on the results obtained in our study, calcite and dolomite particles can be expected to act as slow-release sources of Ca^+2^ and Mg^+2^ ions in solution when the environmental conditions change the dissolution–crystallisation dynamics of fertilizer drop deposits formed on the leaf surfaces after foliar spraying. Factors such as increased relative humidity and lower temperatures overnight can facilitate the continuous dissolution of calcite particles (as calcite is more soluble at low temperatures) to reach equilibrium in the Ca solution, hence acting as a slow-release Ca source. However, future investigations should focus on characterizing with more detail the foliar absorption synergy between mineral particles and essential element cations, taking into account factors such as the combination of different cations in solution, the use of other types of mineral nano-particles (e.g., sulphates), and the addition of adjuvants.

## 4. Conclusions

In this study, trials were carried out to evaluate the absorption rate of micro- and nano- Ca-carbonate particles alone or as nutrient spray adjuvants. The addition of Ca-particles generally increased the absorption of Ca, K, and Mg supplied as soluble salts, but limited the rate of uptake of Zn. The solubility of Ca-carbonate particles in combination with or without soluble essential element salts was evaluated via geochemical modelling. This approach helped to explain to some extent the foliar absorption results obtained with various plant species. Foliar absorption experiments showed that both calcite and dolomite particles used as formulation additives, favoured the uptake of Ca supplied as CaCl_2_, but had a limited capacity to deliver Ca and Mg to the foliage when sprayed alone. The results provide evidence for the major potential of mineral micro- and nano-particles to be used as environmentally friendly foliar agrochemical adjuvants. The improved efficacy of soluble essential element salts in combination with mineral particles may help to reduce fertilizer doses and limit the environmental impact of fertilization in plant production systems, hence contributing to a circular economy. However, more trials will be required for assessing mineral particle performance after foliar application, including potential leaf surface-related phenomena and physiological and metabolic plant responses to foliar fertilizer treatments containing mineral micro- and nano-particles.

## 5. Materials and Methods

### 5.1. Chemicals and Foliar Formulations

Carbonate mineral samples were supplied by Omya International AG (Switzerland). Calcite samples of marble origin with two different particle size distributions were examined: micro-particles (named MicroCal for simplicity) and nano-particles (named NanoCal). MicroCal was provided as a dry powder, while NanoCal was delivered as a slurry (a concentrated nano-particle suspension). For trial development, the concentration of calcite particles was fixed to 2% by weight on the basis of preliminary trials. In selected experiments, dolomite (CaMg(CO_3_)_2_) micro-particles (MicroDol) and nano-particles (NanoDol) were evaluated for comparison of the results obtained with calcite particles. Nano-particles were prepared according to a top-down approach starting from the raw, micro-sized mineral powders produced via a water-based milling process using a bead mill (MicroMedia TM P1, Bühler, Uzwil, Switzerland) equipped with beads (Draison YUP) of 0.1 mm. The volume median particle size of MicroCal and NanoCal is 0.9 µm and ca. 75 nm, respectively. The volume median particle size of MicroDol and NanoDol is 2.3 µm and ca. 50 nm, respectively. Particle size distributions were measured using laser light scattering (Mastersizer 3000, Malvern, Worcestershire, UK) and dynamic light scattering (Zetasizer Nano ZS, Malvern) techniques for micro- and nano-particles, respectively.

All foliar treatments contained 0.075% Break Thru S240 (Oxirane, methyl-, polymer with oxirane, mono [3-[1,3,3,3-tetramethyl-1-[(trimethylsilyl)oxy]disiloxanyl]propyl] ether, AlzChem Trostberg GmbH, Trostberg, Germany) surfactant, for reducing the surface tension to 21.03 ± 0.95 mN m^−1^ (n = 30). The concentration of 0.075% was selected on the basis of the high phytotoxicity risk of this organosilicon surfactant.

The effect of supplying salts of key nutrients (i.e., Ca, Mg, K, or Zn) as active ingredients, either alone or in combination with mineral particles, was also evaluated by spraying 150 mM CaCl_2_ (Sigma-Aldrich, St. Louis, MO, USA), 150 mM KNO_3_ (Sigma-Aldrich), 150 mM MgSO_4_ (Sigma-Aldrich), and 2 mM ZnSO_4_ (Sigma-Aldrich) solutions/suspensions onto model plant species. Formulations (always including a surfactant) containing 2% calcite, sometimes 2% dolomite micro- or nano-particles, and the nutrient element salts described above were sprayed onto the foliage of different plant species as described below.

### 5.2. Geochemical Formulation Modelling

Geochemical simulations were carried out for predicting the behaviour of calcite and dolomite particles in aqueous suspensions/solutions. The main focus was to calculate the Ca concentration and pH of the solutions in equilibrium with calcite particles [45]. These simulations were performed using PHREEQC code and the PHREEQC database [46]. Parameters for zinc sulphates and magnesium carbonates (both anhydrous and hydrated) were taken from the minteq database, and parameters for CaCl_2_ were taken from the llnl database in order to complete the PHREEQC database. All simulations were conducted at 25 °C (the approximate temperature in the laboratory) and without taking into account the effect of the surfactants on the chemical speciation, because their stability constants are not available in the existing databases. In addition, all simulations were carried out considering that the solutions are in equilibrium with the atmospheric CO_2_ and without considering the effect of the atmospheric CO_2_.

### 5.3. Foliar Application Trials

Foliar absorption experiments were carried out both with wettable and unwettable model plant species. In general, foliar sprays were supplied in the morning to benefit from stomatal opening, lower temperature and increased relative humidity. Plants were irrigated the day before foliar spraying and had an adequate nutritional and water status at the time of foliar treatment. All foliar treatments were prepared immediately before foliar application to avoid interaction between formulation components. Leaves were harvested 1 day after spraying and were taken to the lab for washing and further processing before mineral element determination as described below.

Foliar application experiments with sunflower (*Helianthus annuus* L), basil (*Ocimum basilicum* L.), kale (*Brassica oleracea* L. var. sabellica), and Swiss chard (*Beta vulgaris* L. var. cicla) were performed out-of-doors during the summer and autumn at Huertos Comunitarios La Comarca (UTM 30N; 572565, 4435260), Cuenca (Spain).

For evaluating the absorption of calcite particles together with common essential element salts, approximately 3-month-old sunflower and basil plants of homogeneous size and healthy state were sprayed. Sunflower leaves are wettable and have different kinds of trichomes, [47] alike basil leaves [48]. Before the experiments were developed, plants were carefully selected and separated into groups of 3 individuals per treatment. Plants were kept in the open field during the summer, with day and night temperatures ranging between 18 to 35 °C and R.H. from 20 to 85%. In these experiments, MicroCal and NanoCal were applied alone or combined with CaCl_2_, MgSO_4_, KNO_3_, or ZnSO_4_ solutions and 0.075% surfactant. For assessing the rate of foliar penetration of the different foliar formulations, solutions/suspensions were sprayed onto the foliage plants until run–off between 8 to 10 h later (under approximately 50–70% R.H. and 18–22 °C), the roots being protected with aluminium foil to prevent root absorption.

Experiments to evaluate the effect of calcite versus dolomite minerals as Ca formulation adjuvants were carried out with unwettable kale [49] and wettable Swiss chard [50] approximately 3-month-old plants grown in the same experimental plot described above. Again, plants of uniform size and healthy state were selected and labelled in groups of 3 before supplying the foliar treatment. During the foliar spray experiment in autumn, daily environmental conditions varied between 0 to 26 °C and 22 to 96% RH. Foliar sprays contained micro and nano particles of carbonate (named MicroCal, NanoCal) or dolomite (called MicroDol or NanoDol) together with 0.075% Break Thru surfactant in the presence or absence of CaCl_2_ (concentrations are indicated above). Sprays were supplied to the foliage of Swish chard and Kale plants until run–off between 10 to 11 h later (under approximately 50% R.H. and 15 °C) after having protected the roots with aluminium foil for preventing root absorption. Leaves were collected after one day and transported to the laboratory for processing prior to mineral element determination.

The effect of leaf re-wetting via sprinkler irrigation after foliar application was evaluated under greenhouse conditions using unwettable cauliflower plants (*Brassica oleracea* L. var. botrytis) as model plant species [12]. Experiments were performed in autumn at the facilities of the School of Forest Engineering, Technical University of Madrid (UTM 30N; 4477096, 438256). To assess the rate of foliar Ca absorption of calcite particles (MicroCal or NanoCal) supplied alone or in combination with 150 mM CaCl_2_ with or without a surfactant (0.075% Break Thru as described above), foliar treatments were sprayed in the morning (from 8 to 9 a.m.), after covering the base of plants with Al-foil for avoiding root Ca uptake after foliar spray run-off. Half of the treated plants were subjected to a re-wetting–drying cycle 1 day after foliar spraying. The re-wetting process involved a 10 min sprinkler irrigation period (beginning at 11 a.m.). Leaves were collected 1 day after foliar application and 1 day after subjecting a group of foliar-sprayed plants to a process of re-wetting via the described sprinkler irrigation treatment. Harvested leaves were consequently taken to the laboratory and thoroughly washed prior to mineral element analysis, as described below.

### 5.4. Tissue Analysis

At the end of the experimental period (i.e., 1 week for cauliflowers and 1 day after the application of foliar treatments for the rest of the plant species), leaves were cut, separated, and thoroughly washed in an acidulated (0.1 N HCl) 0.1% detergent (Fairy, P&G) solution, also scrubbing the leaf surfaces with the fingers. Plant tissues were then rinsed twice in tap water and then in distilled water. Clean tissues were subsequently oven-dried at 70 °C for two days, weighed and ground to powder, prior to mineral element determination after dry-ashing. The concentration of nutrients in plant tissues was determined by inductively coupled plasma (ICP, Perkin-Elmer, Optima 3000, Waltham, MA, USA), according to UNE-EN ISO/IEC 17025 standard for calibration and testing laboratories (CEBAS-CSIC Analysis Service, Murcia, Spain).

### 5.5. Data Analysis

Statistical analyses were carried out using IBM SPSS Statistics 23.0 programme (IBM Corp., Armonk, NY, USA). Interfacial surface tension values and tissue mineral element concentrations were analysed via one-way analysis of variance (ANOVA). Tukey HSD tests were performed for estimating differences between factors when F-values were significant (*p* < 0.05).

## Figures and Tables

**Table 1:** Calculated Ca concentration in the formulations after reaching equilibrium between the solution and calcite particles (at 25 °C). Solution performance was estimated in equilibrium with atmospheric CO_2_ (with CO_2_) and without considering the influence of atmospheric CO_2_. The calculated pH of the initial solutions in equilibrium with carbonate particles (with and without considering CO_2_) is also shown.

Compound	Solution	pH_initial_	[Ca] (mmol L^−1^)		pH_equilibrium_	
			Without CO_2_	With CO_2_	Without CO_2_	With CO_2_
Calcite (CaCO_3_)	Pure water	7.0	0.12	0.49	9.9	8.3
	150 mM CaCl_2_	6.9	151.63	151.72	8.6	7.3
	150 mM MgSO_4_	6.7	0.88	1.42	9.4	8.4
	150 mM KNO_3_	7.0	0.24	0.76	10.0	8.4
	2 mM ZnSO_4_	6.0	1.26	1.28	8.3	8.1
Dolomite (CaMg(CO_3_)_2_)	Pure water	7.0	0.08	0.30	10.0	8.36
	150 mM CaCl_2_	6.9	151.73	151.89	9.3	7.9

**Table 2:** Cauliflower leaf Ca concentrations, 1 week after spraying water or several Ca-containing formulations. Plants were measured after 1 foliar treatment (no re-wetting) and after an additional 10 min sprinkler irrigation cycle (re-wetting). Data are means ± standard deviations (S.D., N = 3). Different letters indicate homogenous groups according to Tukey’s HSD test (*p* < 0.05).

	Tissue [Ca] (g 100 g^−1^)	
Treatment	No Re-Wetting	Re-Wetting
Water	0.79 ± 0.07 a	-
MicroCal	0.83 ± 0.13 a	0.75 ± 0.06 a
MicroCal + CaCl_2_	1.14 ± 0.10 c	1.13 ± 0.27 b
NanoCal	0.81 ± 0.06 a	0.82 ± 0.05 a
NanoCal + CaCl_2_	1.42 ± 0.32 d	1.39 ± 0.13 c
CaCl_2_	0.98 ± 0.07 b	1.29 ± 0.09 b

## Data Availability

The raw data supporting the conclusions of this article will be made available by the authors, without undue reservation.
